# Relative affordability of heated tobacco products and cigarettes: a cross-country comparative analysis of 55 countries

**DOI:** 10.1186/s12954-026-01509-8

**Published:** 2026-08-06

**Authors:** Giorgi Mzhavanadze

**Affiliations:** 1 Knowledge-Action-Change, 5 Heatherfold Way, Pinner, HA5 2LG UK; 2 Healthy Initiatives (GO Zdorovi Iniciativi), 36 Rustaveli Street, Kyiv, 01033 Ukraine

**Keywords:** Heated tobacco products, Cigarettes, Affordability, Relative income price, Tobacco taxation, Harm reduction, Tobacco control.

## Abstract

**Background:**

Since their introduction in 2014, heated tobacco products (HTPs) have entered a growing number of countries. However, few studies have systematically compared their affordability with that of cigarettes or examined differences in access across income groups. This study assessed the affordability of HTPs relative to combustible cigarettes with a focus on socioeconomic disparities.

**Methods:**

Using publicly available data from the World Health Organization, World Bank, and industry sources, we compared retail prices, affordability, and tax shares across 55 countries with complete analytical data. Affordability was measured as the share of GDP per capita required to purchase 100 packs. Income distribution data were used to assess affordability across income quintiles.

**Results:**

HTP sticks were more expensive than the most-sold cigarette brand in 23 countries and more expensive than the cheapest cigarette brand in 44 countries. In all 9 lower-middle-income countries and 10 of 16 upper-middle-income countries, HTPs were more expensive than the most-sold cigarette brand; in 24 of 30 high-income countries, they were cheaper. HTPs were not marketed in low-income countries. In lower-middle-income countries, purchasing 100 packs required 7.9% of GDP per capita for HTP sticks, compared with 5.3% for the most-sold cigarette brand and 3.5% for the cheapest brand. In high-income countries, the corresponding values were 1.6%, 1.9%, and 1.5%, respectively. For the lowest-income quintile across countries, purchasing 100 packs of HTPs required 9.1% of annual income, compared with 7.9% for the most-sold cigarette brand; for the highest-income quintile, the figures were 1.4% and 1.3%. Including device costs increased annual HTP use costs by 9.2–35.3%.

**Conclusions:**

HTPs were often less affordable than cigarettes, particularly in lower-income countries and among lower-income population groups. This pattern was not explained by higher taxes on HTP sticks; in most countries, HTPs had lower tax shares than cigarettes, but this did not consistently translate into lower retail prices. Assessments of HTP affordability should therefore consider observed consumer prices, upfront device costs, and within-country income differences, rather than tax rates alone.

**Supplementary Information:**

The online version contains supplementary material available at https://doi.org/10.1186/s12954-026-01509-8.

## Background

Since their introduction in 2014, heated tobacco products (HTPs) have become a significant and rapidly growing category within the global nicotine market. Initially launched in Japan and Italy, HTPs are now marketed in at least 70 countries as of early 2025, according to industry data [1, 2]. This expansion has been driven by substantial research and development investment, marketing, and efforts to secure favourable regulatory and tax treatment, primarily by major tobacco companies [1, 3]. Since 2020, the global market value of HTPs, including both consumables and heating devices, has surpassed that of nicotine vaping products, driven by rising prices and growing consumer adoption [3]. However, this market-value advantage does not translate into a larger user base. Recent global estimates indicate that HTP use remains substantially lower than vaping in terms of user numbers: approximately 49 million HTP users in 2024 compared with 129 million vapers in 2025 [4, 5].

Market data suggest that the highest HTP growth rates have been observed in Europe and Asia. In some countries, HTP uptake appears close to one-to-one substitution for cigarettes, such as Hungary, South Korea, Italy, and Germany, whereas in others cigarette sales are declining faster than HTP uptake, such as Slovakia, Japan, and Greece [3].

However, this expansion has been uneven across countries with different income levels. As of April 2025, HTPs were available in at least 40 high-income countries (HICs), 21 upper-middle-income countries (UMICs), and only 9 lower-middle-income countries (LMICs) [2]. Notably, they were not marketed in any low-income countries (LICs), highlighting a significant disparity in access based on national income level.

The public health relevance of these access disparities depends partly on the role that HTPs may play in tobacco harm reduction. This study is positioned within the public health framework of tobacco harm reduction, which focuses on reducing exposure to the harmful constituents of combustible tobacco among adult smokers who are unable or unwilling to quit nicotine use. The principle of harm reduction is based on the understanding that the most severe health consequences of smoking arise primarily from toxicants produced during tobacco combustion, rather than from nicotine itself. HTPs operate by heating tobacco to a temperature sufficient to release a nicotine-containing aerosol, but without burning it. This process results in fewer harmful chemicals than tobacco smoke [6] and substantially reduces the formation of many harmful and potentially harmful constituents [7]. Clinical studies have shown that smokers who switch exclusively to HTPs have significant reductions in exposure to several toxicants [8–12]. Switching to HTPs also eliminates the elevated exhaled carbon monoxide levels associated with smoking, with rapid normalization observed after switching [13, 14]. Recent research further suggests that, after adjustment for prior smoking history, the odds of chronic obstructive pulmonary disease are significantly lower among exclusive HTP users than among cigarette smokers [15].

Despite this growing body of evidence, both the absolute risk of HTP use and the magnitude of relative risk reduction compared with smoking remain subjects of ongoing research. Reviews have noted limitations in the evidence base, including short follow-up, heterogeneous study designs, frequent industry affiliation, and limited data on clinical outcomes [16, 17]. These limitations justify caution but should not be interpreted as evidence that HTPs and cigarettes have equivalent risk. Rather, the remaining uncertainty concerns the magnitude and distribution of any risk reduction, including how this varies by disease outcome and by real-world patterns of use such as complete switching, dual use, partial substitution, or relapse to smoking.

Evidence on population-level health effects remains more limited and is concentrated largely in Japan, where HTP uptake has been unusually rapid. Interrupted time-series analyses have reported reductions in chronic obstructive pulmonary disease (COPD) and ischemic heart disease hospitalisations after HTP introduction [18]. More recent evidence from a national cardiovascular registry found no significant change in total acute coronary syndrome hospitalisations after HTP introduction but reported downward trend changes among adults aged 20–49 years, among smokers, and in areas with higher HTP prevalence [19]. These findings are compatible with the hypothesis that displacement of combustible cigarette smoking by HTP use may reduce some smoking-related outcomes, but they remain observational and cannot establish causality.

This paper does not assess the health outcomes of HTPs. Instead, it focuses on whether HTPs are economically accessible to adult smokers across countries and income groups. Ensuring that potentially less harmful alternatives are accessible across socioeconomic groups is important for health equity [20]. If safer alternatives are not affordable for the populations most burdened by smoking-related diseases, often those in lower socioeconomic groups, their potential to reduce harm at a population level is severely limited [21–24].

In this context, the relative price of HTPs compared with cigarettes becomes a critical determinant of consumer behaviour. Economic evidence shows that raising cigarette prices through taxation reduces smoking prevalence, despite cigarette demand being inelastic [25–28]. By contrast, emerging research suggests that HTP demand is more price-sensitive and that these products function as economic substitutes for cigarettes [29, 30]. It should be noted that alongside price, consumer uptake is also shaped by perceived relative health risks, peer influence, motivations to quit or cut down, convenience, and product availability [31, 32].

Previous studies found that HTPs, despite benefiting from lower tax rates, were still priced higher than cigarettes in many countries [33]. The substantial upfront cost of purchasing a heating device has also been identified as a significant financial barrier that may discourage smokers from switching [34]. Furthermore, these comparisons focus on factory-made cigarettes. In some markets, local, traditional, illicit, informal, or roll-your-own products may provide lower-cost combustible alternatives.

Building on this literature, this paper moves beyond cross-country comparisons of average affordability by focusing explicitly on income-related disparities. It also discusses how tax structures and market pricing may shape access. By focusing on economic equity, this paper provides evidence to guide fiscal and market interventions that could enhance the harm reduction potential of HTPs. To achieve this, we conducted a cross-country analysis of HTP affordability relative to cigarettes, incorporating both national and within-country income differences, as described in the Methods section below.

## Methods

This cross-sectional study compared the affordability of HTPs and combustible cigarettes in countries where HTPs were legally available and all data required for analysis could be obtained. Countries were included only if complete information was available on HTP stick prices, cigarette prices, tax shares, GDP per capita, and income distribution. The analysis was stratified by national income level and within-country income quintile.

Data were compiled from public and industry sources. Retail prices and tax shares for the most-sold, premium, and cheapest cigarette brands, together with the most-sold brand of HTP sticks, were obtained from the World Health Organization’s (WHO) Global Health Observatory for 2022 [35]. HTP heating device prices were taken from industry-reported retail selling price data as of April 2024 [36]. Country-level macroeconomic indicators for 2022, including gross domestic product (GDP) per capita and income distribution by quintile, were obtained from the World Bank; national income classifications followed the World Bank fiscal year 2024 groupings [37, 38].

After applying these inclusion criteria, the final analytical sample comprised 55 countries. HTP-available markets were excluded mainly because WHO-reported HTP stick prices and/or HTP tax shares were unavailable; several also lacked complete cigarette price data. The analysed countries and available source values are reported in Supplementary Table S1.

The primary affordability measure was the Relative Income Price (RIP), defined as the percentage of annual GDP per capita required to purchase 100 packs of a given product, with higher RIP values indicating lower affordability [39]. RIP was calculated for the general population and for each income quintile. To account for the upfront cost of HTP use, the analysis also estimated device-inclusive HTP affordability. This was calculated by adding a one-year heating device cost to the cost of 100 packs of HTP sticks. The lowest- and highest-priced devices reported in April 2024 were used to define lower and upper device-cost scenarios. This approach applies the common 100-pack RIP denominator across all countries and does not model country-specific differences in HTP consumption intensity. Descriptive analysis was supplemented with ordinary least squares linear regression to examine whether HTP stick prices were associated with the price of the most-sold cigarette brand, the tax-share differential between cigarettes and HTP sticks, and GDP per capita. The regression model was specified as follows:

$$P_{{\mathrm{HTP}}i}=\beta_0+\beta_1P_{{\mathrm{CIG}}i}+\beta_2TD_i+\beta_3GDP_{{\mathrm{PC}}i}+\varepsilon_i$$

where P_HTPi_ is the retail price of the most-sold HTP stick brand in country *i*; P_CIGi_ is the retail price of the most-sold cigarette brand in US dollars; TD_i_ is the cigarette total tax share minus the HTP total tax share, expressed in percentage points; GDP_PCi_ is GDP per capita expressed in units of US$1,000; and ε_i_ is the error term.

All calculations and statistical analyses were conducted in Microsoft Excel (Microsoft 365; Microsoft Corporation, Redmond, WA, USA).

## Results

### Cross-country price comparison

Across the 55 countries analyzed, HTP sticks were more expensive than the most-sold brand of cigarettes in 23 countries and more expensive than the cheapest brand of cigarettes in 44 countries. This pattern varied by national income level: HTP sticks were more expensive than the most-sold cigarette brand in all 9 LMICs and 10 of the 16 UMICs in the sample. In contrast, among the 30 HICs, HTPs were less expensive than the most-sold cigarette brand in 24 countries.

**Fig. 1:**
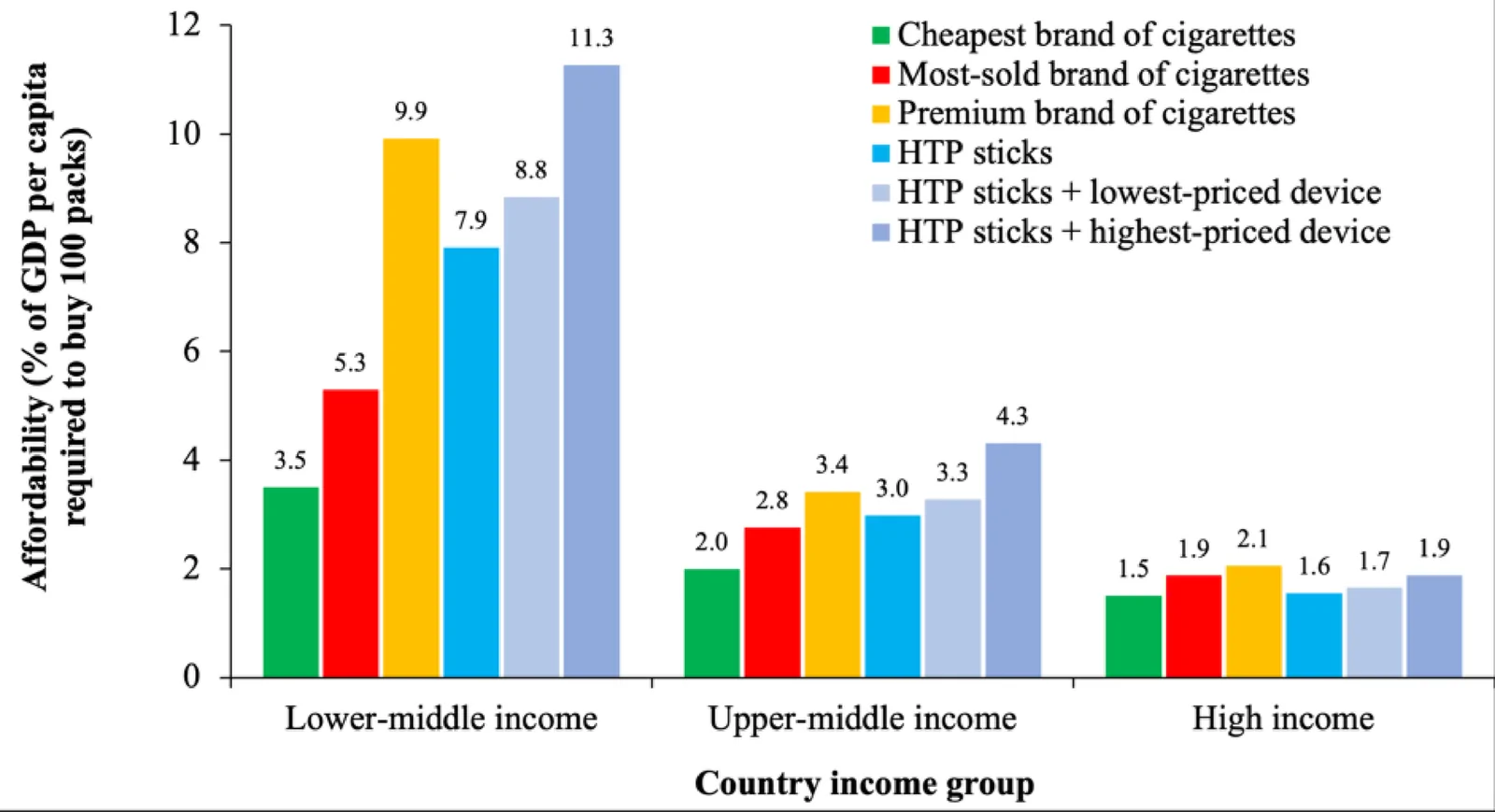
Price Comparison Between Heated Tobacco Products and Cigarettes Across 55 Countries, 2022. Each point represents one country. Red circles plot the price of heated tobacco product (HTP) sticks against the price of the most-sold cigarette brand; green diamonds plot HTP stick price against the cheapest cigarette brand; yellow diamonds plot HTP stick price against the premium cigarette brand. Prices are shown in US$ per 20-stick pack. The 45° parity line indicates equal prices; points above the line denote HTPs priced higher than the cigarette brand, and points below the line denote HTPs priced lower. HTP device costs are not included.

Descriptive analysis indicated a negative relationship between absolute cigarette prices and the price differential between HTPs and cigarettes: in countries with higher cigarette prices, HTPs were more likely to be the cheaper option, and vice versa (Fig. 1).

Multiple linear regression supported this descriptive pattern. After adjusting for tax differential and GDP per capita, each US$1 increase in the most-sold cigarette price was associated with a US$0.51 higher HTP stick price (95% CI: 0.4526–0.5695; *p* < 0.001). This implies that, on average, a US$1 increase in cigarette price was associated with an approximately US$0.49 increase in the cigarette-HTP price gap, making HTPs relatively more likely to be cheaper than cigarettes in higher-cigarette-price markets. GDP per capita was also positively associated with HTP stick price (coefficient per US$1000: 0.0076; 95% CI: 0.0014–0.0137; *p* = 0.0166). The tax-share differential between cigarettes and HTP sticks was not statistically significant (Table 1).

**Table 1:** Linear regression results for heated tobacco products prices

Variable	Coefficient	Standard error	t-statistic	*P*-value	95% CI
Intercept	1.3108***	0.1625	8.0654	< 0.0001	0.9845–1.6371
Tax differential	0.0052	0.0044	1.1926	0.2385	-0.0036–0.0141
Most-sold cigarette price	0.5111***	0.0291	17.5458	< 0.0001	0.4526–0.5695
GDP per capita	0.0076**	0.0031	2.4774	0.0166	0.0014–0.0137

Ordinary least squares regression of HTP stick price (US$ per 20-stick pack) as the dependent variable, using 2022 cross-country data (*n* = 55). Independent variables were the tax-share differential between cigarettes and HTP sticks (percentage points), the price of the most-sold cigarette brand (US$), and GDP per capita (US$1000). Adjusted R2 = 0.9221. Coefficients are rounded to four decimal places. ***p < 0.001, **p < 0.05. CI: confidence interval; GDP: gross domestic product

### Affordability by national income level

**Fig. 2:**
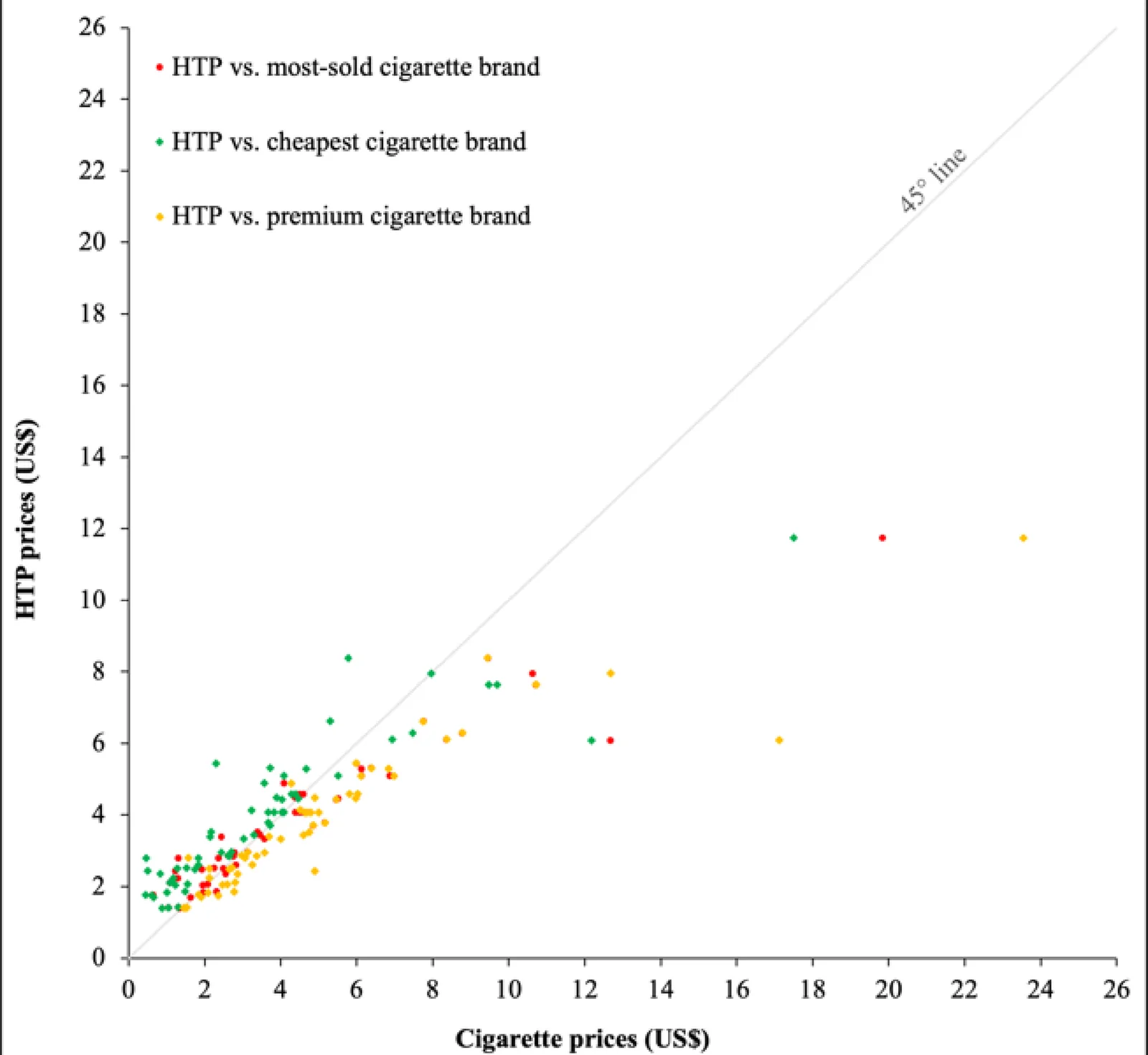
Affordability of cigarettes and heated tobacco products by country income group, 2022. Bars show affordability as the Relative Income Price (RIP), the percentage of gross domestic product (GDP) per capita required to purchase 100 packs. Product categories include the cheapest, most-sold, and premium cigarette brands; heated tobacco product (HTP) sticks alone; HTP sticks plus the lowest-priced device; and HTP sticks plus the highest-priced device. Device costs are annualized over one year. Country groups are lower-middle-, upper-middle-, and high-income.

Affordability varied substantially across income groups (Fig. 2). In LMICs, purchasing 100 packs required 7.9% of GDP per capita for HTP sticks, compared with 5.3% for the most-sold cigarette brand, 3.5% for the cheapest brand, and 9.9% for the premium brand. In UMICs, the corresponding RIP values were 3.0% for HTP sticks, 2.8% for the most-sold cigarette brand, 2.0% for the cheapest brand, and 3.4% for the premium brand. In HICs, HTP sticks required 1.6% of GDP per capita, compared with 1.9% for the most-sold cigarette brand, 1.5% for the cheapest brand, and 2.1% for the premium brand. Across all groups, the cheapest cigarette brands remained the most affordable option. The affordability gap between HTPs and cigarettes narrowed as national income levels increased, making HTPs relatively more accessible in wealthier countries. No LICs in the dataset had HTP availability.

### Affordability by income quintile

**Fig. 3:**
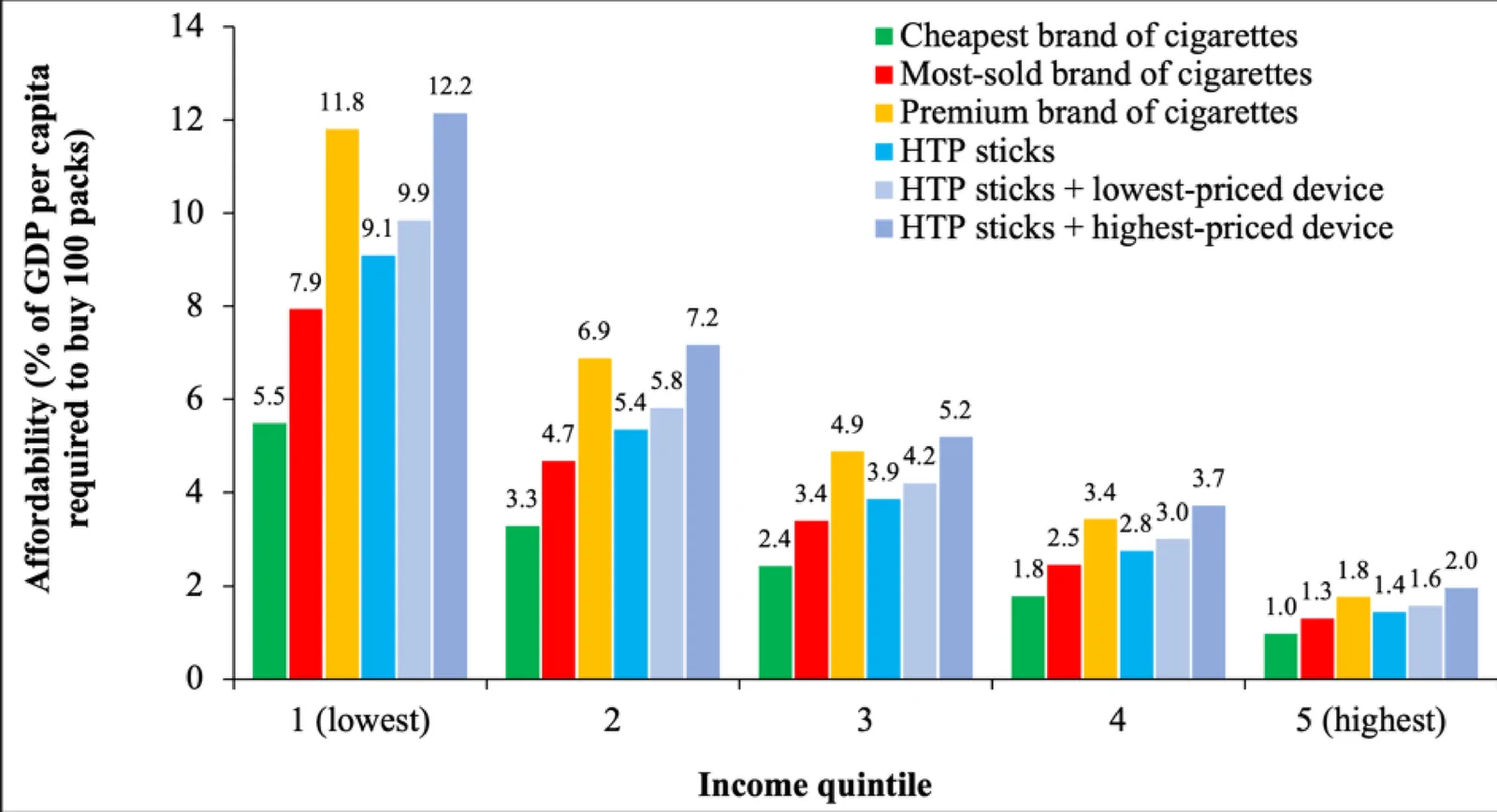
Affordability of Cigarettes and Heated Tobacco Products Across Income Quintiles, 2022. Bars show affordability as the Relative Income Price (RIP), the percentage of gross domestic product (GDP) per capita required to purchase 100 packs, for income quintiles within countries (Q1 = poorest 20%, Q5 = richest 20%). Values reflect averages across the 55-country sample. Product categories include the cheapest, most-sold, and premium cigarette brands; HTP (heated tobacco product) sticks alone; HTP sticks plus the lowest-priced device; and HTP sticks plus the highest-priced device. Device costs are amortized over one year

Within countries, affordability gaps were most pronounced for lower-income groups (Fig. 3). For the lowest-income quintile across all countries, purchasing 100 packs of HTP sticks would require 9.1% of annual income, compared with 7.9% for the most-sold cigarette brand. Among the highest-income quintile, the corresponding values were 1.4% and 1.3%, respectively. Thus, HTP sticks remained less affordable than the most-sold cigarette brand across income quintiles, although the absolute burden was concentrated among lower-income groups.

### Impact of device costs

Including the annual cost of HTP devices increased the total annual cost of HTP use by 9.2% to 35.3%, depending on device model. When the cost of the cheapest device was included, HTPs became more expensive than the most-sold cigarette brand in 39 of the 55 countries, including all 9 LMICs and 14 of the 16 UMICs. When the most expensive device was factored in, HTPs were more expensive in 53 of 55 countries. The only exceptions where cigarettes remained costlier even with the highest-priced device were the United Kingdom and New Zealand.

### Tax burden analysis

The higher price of HTPs was not explained by higher tax shares in this cross-sectional analysis. HTPs had a lower or equal tax burden (measured as the share of total taxes in retail price) than cigarettes in 52 of the 55 countries studied. The average tax gap between cigarettes and HTPs was 24.3% points. This tax-share gap was largest in HICs (27.4% points) and smallest in LMICs (16.5% points). In the regression model, the tax differential was not statistically associated with HTP stick price (Table 1).

## Discussion

This study provides cross-country evidence that HTPs are often less affordable than cigarettes, particularly in lower-income settings and among lower-income population groups. Although HTPs are frequently discussed as potentially lower-risk alternatives to combustible cigarettes, their public health relevance depends in part on whether adult smokers can realistically access them [6, 8–11, 13–15, 40, 41]. Our findings suggest that, in many markets, price and affordability may limit such access. This is especially important because smoking-related disease is socially patterned, with lower-income groups generally bearing a larger burden of tobacco-related harm [20–24].

The affordability gap was not explained by higher tax burdens on HTP sticks. In 52 of the 55 countries included in the analysis, the measured total tax share in the retail price was lower for HTP sticks than for cigarettes. However, this tax advantage was not consistently reflected in lower retail prices. This finding is consistent with previous cross-country evidence showing that HTPs have often received preferential tax treatment compared with cigarettes, but that tax differences do not necessarily translate into equivalent differences in consumer prices [29, 33]. The distinction is important for policy interpretation. A lower tax rate may create fiscal space for lower HTP prices, but the final retail price also depends on manufacturer pricing, profit margins, market structure, brand positioning, and consumer demand.

Several mechanisms may help explain why HTPs remain relatively expensive despite lower tax shares. First, HTPs differ from cigarettes in their production and commercial structure. HTP systems combine processed tobacco consumables with proprietary electronic heating devices, chargers, and related support infrastructure [7]. Industry reports substantial cumulative investment in smoke-free products, including product development, scientific assessment, and commercialisation [42]. These reported investments do not demonstrate that higher retail prices are cost-based, but they suggest that HTPs involve fixed costs and device-related requirements that differ from mature cigarette production. Conventional cigarettes, by contrast, are produced through long-established, high-volume manufacturing systems and have been shown to remain highly profitable despite substantial excise taxation [43]. Part of the HTP price premium may therefore reflect fixed-cost recovery, device subsidies, product servicing, and manufacturing complexity. These factors may create a higher practical price floor for HTPs than for cigarettes, although the present data cannot separate production costs, R&D recovery, taxes, wholesale and retail margins, promotional discounts, or manufacturer profit.

This interpretation is also relevant for understanding relative affordability. If HTP prices cannot fall below a certain level in many markets, then HTPs may become more affordable than cigarettes mainly where cigarette prices are sufficiently high. In other words, the relative affordability of HTPs depends not only on whether HTPs receive preferential tax treatment, but also on whether cigarettes are priced high enough to make switching financially attractive. The examples of high-cigarette-price markets in our sample, including the United Kingdom and New Zealand, are consistent with this pattern.

Second, HTP pricing may reflect portfolio and brand-positioning strategies. Earlier cross-country evidence found that HTP sticks were commonly priced close to premium cigarette brands rather than to lower-priced cigarette brands [33]. If HTP consumables are positioned against premium cigarettes, they may appear expensive when compared with the most-sold or cheapest cigarette brands, particularly in countries where low-price cigarette segments remain large. Under this interpretation, HTPs may function as a premium tier within a broader nicotine product portfolio rather than as the lowest-cost substitute for smoking. Our study did not compare HTPs with e-cigarettes, nicotine pouches, or other non-combustible nicotine products, and therefore cannot assess whether lower-priced non-combustible alternatives were available in the same markets.

Third, the relative affordability of HTPs is shaped not only by HTP prices, but also by cigarette prices. In our data, cigarette tax shares were lower on average in lower-middle-income countries than in high-income countries, and higher cigarette prices were associated with greater relative affordability of HTPs. This finding is consistent with the broader tobacco-tax literature showing that cigarette affordability is an important determinant of consumption and that tax policy remains one of the strongest tools for reducing smoking [25–28, 39]. It also suggests that preferential taxation of HTPs may have limited effect on affordability if cigarette prices remain low. However, the relationship between cigarette taxes, HTP taxes, and product substitution is complex. A risk-proportionate tax approach has been proposed to align nicotine-product taxation with differences in product risk [44]. Recent evidence also suggests that HTP demand is price-sensitive [45]. This implies that differential taxation should be evaluated against observed retail prices and consumer responses, not only statutory tax rates.

Fourth, affordability is only one component of access. National regulation determines whether HTPs and other non-combustible nicotine products can be legally sold, which product features are permitted, and what marketing or retail restrictions apply. International guidance generally recommends strict regulation of novel and emerging nicotine and tobacco products, while some jurisdictions permit access under product authorisation, age restrictions, marketing controls, or modified-risk regulatory pathways [46]. These regulatory differences may affect product availability, competition, compliance costs, retail channels, and final prices. Our analysis does not quantify regulatory barriers, product-authorisation costs, flavour restrictions, or retail-channel constraints, but these factors should be considered when interpreting affordability as one part of a wider access environment.

Beyond consumer access, the findings also suggest caution in interpreting industry “transformation” claims. At the same time, profit maximisation and movement into non-combustible products are not mutually exclusive: firms may invest in HTPs because of changing consumer preferences, regulatory pressure, declining cigarette volumes in some markets, and the long-run commercial potential of alternative nicotine products. However, transformation claims should be assessed against verifiable reductions in combustible product sales, not only growth in new product revenues [47–52]. Global claims also require caution because China, the world’s largest tobacco market, is dominated by a state-owned tobacco industry largely outside the control of transnational tobacco companies [53, 54]. For these reasons, our findings should be interpreted as evidence of affordability differences in markets where HTPs are sold, not as proof of corporate motives or as a complete assessment of the global nicotine market.

In addition to HTP stick prices, the upfront cost of heating devices remains a separate affordability barrier. Even when HTP stick prices are close to cigarette prices, the need to purchase a device can increase the initial cost of switching. This may be particularly relevant for lower-income smokers, for whom a device purchase can represent several weeks or months of cigarette expenditure. Device prices may also be affected by promotions, starter kits, discounts, loyalty schemes, and temporary subsidies. As a result, recommended or retail device prices included in the analysis may not fully capture the prices paid by consumers at the point of purchase.

A further equity issue is that our estimates may understate the affordability gap faced by some smokers because the analysis compares HTPs with manufactured cigarettes rather than roll-your-own (RYO) tobacco. RYO tobacco is often cheaper than factory-made cigarettes and is disproportionately used by lower socioeconomic groups [55–58]. For smokers who use RYO tobacco, the financial barrier to switching to premium-priced HTPs is likely to be larger than the comparison with manufactured cigarette prices suggests. Lower-income populations are also more price sensitive and more prone to downward substitution [55–60]. This reinforces the equity relevance of the findings: lower-income smokers may be least able to access higher-priced alternatives, even where those products are legally available.

The main policy implication is that lower HTP tax rates should not be assumed to produce lower consumer prices. Where governments permit HTPs and seek to create financial incentives for switching away from combustible cigarettes, affordability should be monitored using retail prices and income-based measures, not tax rates alone. Preferential tax treatment may have limited effect if lower taxes are not passed through to consumers or if cigarette prices remain low. Conditional tax structures could, in principle, link preferential treatment to observable retail-price differences between HTPs and cigarettes, although such an approach would require careful design, reliable price monitoring, and clear enforcement mechanisms [61, 62]. More interventionist approaches, such as price ceilings or profit-margin regulation, have been discussed in other sectors but would carry risks, including reduced product availability, lower incentives for innovation, administrative complexity, and possible informal-market responses [63–65]. Competition policy may also be relevant: where legally available products are limited to a small number of premium brands, market entry and product diversity may affect prices and affordability [66]. These policy options require empirical evaluation and should be considered within each country’s broader tobacco-control and nicotine-regulation framework.

## Limitations

This study has several limitations. First, the analysis is based on cross-sectional data from 2022 and should be interpreted as a snapshot of market conditions. Tobacco and nicotine product markets are dynamic, with prices, taxes, product availability, and regulation changing over time. The analysis is therefore descriptive and cannot establish causal relationships between tax structures, retail prices, affordability, and consumer behaviour.

Second, the analysis relies on available international price and tax datasets. These sources allow standardized cross-country comparison but may not capture all local price variation, temporary promotions, retailer discounts, online prices, loyalty schemes, bundled starter kits, or informal-market prices. This is particularly relevant for device-inclusive affordability estimates, because device prices may be more heavily affected by promotions than consumable stick prices.

Third, heating device cost estimates are drawn from a single company for 2024. This may introduce some distortion, although this source provided the most complete cross-country device-price data available for the analysis. A comparison of heating device prices between 2024 and 2025 revealed no consistent pattern of price change, suggesting that the figures used are a reasonable approximation of listed device prices [2, 36].

Fourth, the device analysis assumes that one heating device is amortised over one year and spread across 100 packs, equivalent to 2,000 sticks or about 5.5 sticks per day. This assumption was applied uniformly across countries to create a standardized comparison. Actual HTP consumption may vary across countries, products, and user groups. However, the assumption is broadly consistent with available evidence. For example, recent representative survey evidence from Germany reported that current HTP users consumed an average of 7.3 sticks per day [67]. The 2,000-stick annual assumption is therefore somewhat conservative but close to reported real-world use. If average consumption is lower than assumed, the device cost per stick would be higher and vice versa. The device-inclusive affordability estimates should therefore be interpreted as standardized approximations rather than precise country-specific measures of annual user cost.

Fifth, the analysis uses the most-sold HTP stick price reported by WHO. This improves cross-country comparability but may miss lower-priced HTP variants available in some markets. If cheaper HTP consumables are widely available, the affordability gap reported here may overstate the cost barrier faced by some consumers.

Sixth, GDP per capita was used as the primary income indicator. Although this provides a comparable measure across countries, it does not capture disposable income, household expenditure, or within-country income inequality. This limitation was partly addressed by using income quintile data, but more granular household-level data would allow a more precise assessment of affordability by socioeconomic position.

Seventh, the tax-burden measure is based on the share of total taxes in the retail price. This provides a standardized international proxy but does not directly capture statutory excise structures. HTPs and cigarettes may be taxed through specific, ad valorem, or mixed systems, and identical statutory tax rates can produce different tax shares when retail prices differ. This limitation may introduce some imprecision in interpreting the tax gap. However, it does not alter the main descriptive finding that lower measured tax shares for HTPs were not consistently associated with lower consumer prices.

This analysis does not include all nicotine or tobacco products that may be relevant substitutes for cigarettes. E-cigarettes, nicotine pouches, snus, nicotine replacement therapy, and illicit or informal products were outside the scope of this study. This limits the ability to assess the relative affordability of HTPs within a wider product landscape.

Finally, this study does not evaluate the health effects of HTPs or their effectiveness for smoking cessation. Its focus is economic affordability and equity of access. The public health implications of HTP affordability depend on the extent to which smokers switch from combustible cigarettes, patterns of dual use, the relative risks of HTP use compared with smoking, and uptake among nicotine-naive individuals.

## Conclusions

HTPs were frequently less affordable than cigarettes in the countries included in this study, particularly in lower-income settings and among lower-income population groups. This pattern was not explained by higher measured tax shares on HTP sticks; in most countries, HTPs had lower tax shares than cigarettes, but this did not consistently translate into lower retail prices.

These findings suggest that assessments of HTP affordability should be based on observed consumer prices, not tax rates alone. They also highlight the importance of considering upfront device costs and within-country income differences when evaluating access to non-combustible nicotine products. Further research is needed to examine how tax policy, manufacturer pricing, market competition, regulatory conditions, and consumer behaviour interact to shape the affordability and uptake of HTPs across different settings.

## Supplementary Information

Below is the link to the electronic supplementary material.

Supplementary Material 1

## Data Availability

The datasets analysed during the current study are available in the following repositories:WHO Global Health Observatory:o National taxes and retail price for 20 sticks of heated tobacco products [https://www.who.int/data/gho/data/indicators/indicator-details/GHO/national-taxes-and-retail-price-for-20-sticks-of-heated-tobacco-products]. National taxes on a pack of 20 cigarettes [https://www.who.int/data/gho/data/indicators/indicator-details/GHO/gho-tobacco-control-raise-taxes-national-taxes-pack-of-20]. Retail price for a pack of 20 cigarettes [https://www.who.int/data/gho/data/indicators/indicator-details/GHO/gho-tobacco-control-raise-taxes-retail-price-for-a-pack-of-20-cigarettes]. World Bank Data:o GDP per capita (constant national currency) [https://data.worldbank.org/indicator/NY.GDP.PCAP.CN?view=chart]. GDP per capita (current US$) [https://data.worldbank.org/indicator/NY.GDP.PCAP.CD?view=chart]. Income shares by quintile: First [https://data.worldbank.org/indicator/SI.DST.FRST.20?view=chart]. Second quintile [https://data.worldbank.org/indicator/SI.DST.02ND.20?view=chart]. Third quintile [https://data.worldbank.org/indicator/SI.DST.03RD.20?view=chart]. Fourth quintile [https://data.worldbank.org/indicator/SI.DST.04TH.20?view=chart]. Fifth quintile [https://data.worldbank.org/indicator/SI.DST.05TH.20?view=chart]. World Bank country and lending groups classification [https://datahelpdesk.worldbank.org/knowledgebase/articles/906519-world-bank-country-and-lending-groups]. Industry data (Philip Morris International):o Retail selling price database – April 2024 release [https://www.pmi.com/resources/docs/default-source/investor_relation/may_2024---rsps.xlsx]. Retail selling price database – April 2025 release [https://www.pmi.com/resources/docs/default-source/investor_relation/apr25---rsps.xlsx].The compiled dataset used for analysis is provided in Supplementary Table S1.

## Abbreviations

COPD: Chronic obstructive pulmonary disease
GDP: Gross domestic product
HIC: High-income country
HTP: Heated tobacco product
LIC: Low-income country
LMIC: Lower-middle-income country
RIP: Relative income price
RYO: Roll-Your-Own
UMIC: Upper-middle-income country
WHO: World Health Organization

## Declarations

### Use of AI-assisted technologies

The author discloses that AI chatbot ChatGPT was used solely for language editing purposes in the preparation of this manuscript. The model was not used for data analysis, data interpretation, or content generation. All intellectual content, analysis, and conclusions are my own, and I take full responsibility for the integrity and accuracy of the work.
